# Platelet antibody detection assays: a single-laboratory comparison of MAIPA, PIFT, and microsphere-based multiplex assays Pak-Lx

**DOI:** 10.1016/j.htct.2024.06.004

**Published:** 2024-09-07

**Authors:** Thiago Henrique Costa, Carolina Bonet-Bub, José Mauro Kutner

**Affiliations:** Hospital Israelita Albert Einstein, São Paulo, SP, Brazil

**Keywords:** Antibodies, Alloimmunization, Human leucocyte antigens (HLA), Platelet antibody detection, Human platelet antigens (HPA), MAIPA

## Abstract

**Background and Objectives:**

The identification of platelet antibodies is essential for diagnosing and managing conditions such as fetal and neonatal alloimmune thrombocytopenic purpura, post-transfusion purpura, and immune platelet refractoriness. Monoclonal antibody immobilization of platelet antigens (MAIPA) is the standard method for detecting anti-human platelet antigen (HPA) antibodies, while the detection of anti-HLA antibodies once relied on the complement-dependent cytotoxicity method, however advanced technologies such as enzyme-linked immunosorbent assay and Luminex have significantly improved sensitivity and accuracy in identifying these antibodies. Flow cytometry-based techniques (platelet immunofluorescence test - PIFT) and Luminex platform-driven microsphere-based multiplex assays (Pak-Lx) are widely employed in platelet immunology laboratories owing to their remarkable flexibility and versatility. The present study compared the sensitivity, specificity, and concordance of these different serological techniques used in platelet antibody identification.

**Material and Methods:**

One hundred serum samples from patients suspected of immune-mediated platelet disorders were examined. Initially, the samples underwent testing using the MAIPA method. Subsequently, the results were compared with three alternative methods: PIFT and microsphere-based multiplex assays for both HLA and HPA antibodies.

**Results:**

Pak-Lx demonstrated a 94 % agreement with MAIPA, while PIFT had 88 % agreement for HPA antibodies. For HLA antibody detection, Pak-Lx versus DLX had 75 % concordance, MAIPA versus DLX showed 77 %, and PIFT versus DLX displayed an 81 % concordance rate. Remarkably, there were no significant differences in concordance levels between Pak-Lx and PIFT compared to MAIPA and DLX for anti-HPA and HLA antibodies, respectively.

**Conclusion:**

This study found no significant differences in concordance among the tested assays for detecting anti-HPA and anti-HLA antibodies. These data suggest that no single method can detect all clinically important antibodies. Therefore, it is advisable that each laboratory develops customized protocols based on their expertise and employs complementary methods for comprehensive patient assessments.

## Introduction

Platelets express a variety of glycoproteins (GPIIb-IIIa, Ia-IIa, Ib/IX, IV, CD109, and class I human leukocyte antigens (HLA) on their surface. These glycoproteins contain highly polymorphic human platelet antigens (HPA) and HLA.^1^^,^^2^ Polymorphisms can be targeted by the immune system in incompatible individuals and induce the formation of clinically significant antibodies.^3^ The detection and identification of antibodies have an important role in the diagnosis and prevention of complications in neonatal and fetal alloimmune purpura (FNATP), post-transfusion purpura (PTP), and immune platelet refractoriness (IPR).^4^^,^^5^

Currently, several techniques can be used in the detection of specific antibodies against platelets, including monoclonal antibody-specific immobilization of platelet antigen (MAIPA), platelet immunofluorescence test (PIFT) and multiplex assays based on microspheres using the Luminex platform.^5^, ^6^, ^7^

MAIPA was developed by Kiefel et al. in 1987.^6^ It is a laborious technique that utilizes the antigens present on the platelet surface and monoclonal antibodies (MoAbs) to detect human platelet antibodies. The technique requires large volumes of serum, and takes approximately 8–12 h.^6^^,^^8^, ^9^, ^10^ MAIPA is considered the most sensitive and specific technique to detect antibodies directed against the antigens present in glycoproteins. This feature is also valuable for detecting new specificities and antibodies directed at low-frequency antigens when performing a crossmatch between donor and recipient or when using paternal platelets in cases of suspected neonatal alloimmune thrombocytopenia.^11^ The application of MAIPA for detecting antibodies directed against class I HLA system antigens is not a routine due to the significant genetic variability of this system in the population.^11^, ^12^, ^13^

PIFT detects most causes of IgG and IgM binding to platelets, including antibodies against class I HLA antigens, platelet-specific antigens, and autoantibodies.^14^^,^^15^ PIFT exhibits high sensitivity and low specificity; it will detect all antibodies present in the serum simultaneously, including both HPA and class I HLA antibodies, using 50 μL of sample per test. The assay is straightforward and quick, requiring no significant serum volume. However, it is necessary to have a set of fresh or frozen platelets with known HPA and HLA antigens available for the tests. Its most common use is in the screening of platelet antibodies and in matching patients with platelets to be transfused.^13^^,^^16^, ^17^, ^18^

The Luminex-based assay employs polystyrene microbeads infused with a distinctive combination of two fluorescent dyes, both concurrently excited by a red laser at a wavelength of 635 nm. By assessing the emission intensity in both channels, the system can simultaneously identify up to 100 unique beads, each carrying a specific antigen. Antibody detection is accomplished through the utilization of a secondary antibody linked to the reporter fluorophore R-phycoerythrin, which, in turn, is excited by a green laser (532 nm).^19^ Each microsphere is designed to carry a specific platelet glycoprotein, thereby enhancing the precision of the analysis.^10^^,^^19^ It is a technique that is quick, sensitive, and specific, that does not require a large sample volume. The technology of Luminex allows for the simultaneous detection and identification of antibodies directed against HPA (HPA-1, HPA-2, HPA-3, HPA-4, HPA-5, GPIV, and class I HLA) and HLA (class I and class II antigens) in a single run using 10 μL of sample per test.^20^, ^21^, ^22^, ^23^

The present study compares the sensitivity, specificity, and concordance of these different serological techniques used in anti-HPA and anti-HLA antibodies identification.

## Materials and methods

The local ethics committee approved this project under the ethical process number: 50997721.0.0000.0071.

Samples were collected from patients with suspected diagnosis of IPR, FNATP, or PTP evaluated at the Hemotherapy and Cellular Therapy Department of the Albert Einstein Israelita Hospital, São Paulo between 2019 and 2021. As the samples had undergone prior MAIPA testing, they were subsequently assessed using additional techniques, including PIFT and microsphere-based multiplex assays for both HLA (DLX assay) and HPA (Pak-Lx Assay) antibodies. The results obtained from MAIPA were then compared with those from the other three methodologies. The MAIPA test was considered the reference test for the detection of anti-HPA antibodies, and the microsphere-based multiplex assays for HLA was considered the reference test for the detection of anti-HLA antibodies.

### Platelet antibody detection - Monoclonal antibody immobilization of platelet antigens

The MAIPA test was performed according to the procedures described by Kiefel et al.^6^ The cutoff for positive values was defined as the mean of ten negative control samples plus three standard deviations (>0.2).

Monoclonal antibodies against CD41 (GPIIb, Clone P2, Beckman Coulter, Indiana, United States), CD42a (GPIX, clone SZ1, Beckman Coulter, Atlanta, USA), CD49b (GPIIa, Clone Gi9, Beckman Coulter, Atlanta, USA), CD109 (Clone TEA 2/16, Becton Dickinson Immunocytometry Systems, San Jose, CA), and Beta2-microglobulin (MHC-I, clone B1G6, Beckman Coulter, Atlanta, USA) were used to capture the antigens. Moreover, goat anti-mouse IgG (Jackson ImmunoResearch®, West Grove, PA, USA), peroxidase AffiniPure F(ab')₂ and fragment goat anti-human IgG antibodies (*H* + *L* - Jackson ImmunoResearch®, West Grove, PA, USA) were used for fixation and detection.

### Platelet antibody detection - platelet immunofluorescence test

The PIFT test was performed according to the procedure described by Von Dem Borne et al.^14^ A total of 10,000 events were acquired for analysis according to the forward and sideways light scatter characteristics*.* A fluorescence intensity graph was plotted for the analysis. Monoclonal antibodies against human IgG (F(ab')2-goat anti-human IgG Fc secondary antibody, FITC - Life Technologies, Switzerland) were used for detection. The cutoff for positive values was defined as the mean fluorescence intensity (MFI) of ten negative control samples plus three standard deviations (5.85).

### Detection of antibodies by multiplex assay based on microspheres using luminex

The detection and identification tests for anti-HPA (HPA-1, HPA-2, HPA-3, HPA-4, HPA-5, GPIV and HLA class I) antibodies were performed using the Pak-Lx assay kit (Immucor, Norcross, GA, United States). The tests were conducted following the procedure provided by the manufacturer. After the reaction, the MFI of each bead used in the test was measured with the Luminex 200 instrument (Luminex, Austin, Texas, United States). The MATCH IT! antibody software program (Immucor, Norcross, GA, United States) was used for analysis and interpretation.

### Detection of HLA-I antibodies by multiplex assay based on microspheres using the luminex platform

The detection test for anti-HLA class I antibodies was performed using the Lifecodes LifeScreen Deluxe kit (Immucor, Norcross, GA, United States). The test was conducted following the procedure described by the manufacturer. After the reaction, the MFI of each bead used in the test was measured with the Luminex 200 instrument (Luminex, Austin, Texas, United States). The MATCH IT! antibody software program (Immucor, Norcross, GA, United States) was used for analysis and interpretation.

### Statistical analysis

All data were evaluated employing the McNemar test. The sensitivities and specificities of the assays were compared to the results obtained from the MAIPA assay for HPA antibodies and by the LifeScreen Deluxe assay for HLA antibodies.

## Results

One hundred samples were analyzed, 71 (71 %) from women and 29 (29 %) from men with a median age of 51 (range: 1–92) years. Clinically, 82 suffered from IPR, 17 from FNATP, and one from PTP.

The specificities of the identified antibodies were as follows: anti-HLA (*n* = 28; 24.78 %), anti-HPA1a (*n* = 13; 11.5 %), anti-HPA1b (*n* = 2; 1.77 %), anti-HPA2b (*n* = 3; 2.65 %), anti-HPA3a (*n* = 1; 0.88 %), anti-HPA3b (*n* = 1; 0.88 %), anti-HPA5a (*n* = 1; 0.88 %), anti-HPA5b (*n* = 5; 4.42 %), anti-IIb/IIIa (*n* = 1; 1.77 %), anti-Ia/IIa (*n* = 1; 1.77 %), with a negative result in 55 samples (48.67 %). No antibodies were directed against the HPA antigens (2a, 4a, 4b, 15a, 15b and GPIV - Tables 1 and 2).

**Table 1:** Comparison of results obtained by the platelet immunofluorescence test (PIFT) and Pak-Lx methodologies in relation to monoclonal antibody-specific immobilization of platelet antigen (MAIPA).

	MAIPA n (%)		
	Positive	Negative	Total
Pak-Lx			
Positive	40 (40)	2 (2)	42
Negative	4 (4)	54 (54)	58
Total	44	56	100
PIFT			
Positive	42 (42)	10 (10)	52
Negative	2 (2)	46 (46)	48
Total	44	56	100

**Table 2:** Comparison of results obtained by Pak-Lx, platelet immunofluorescence test (PIFT), and monoclonal antibody-specific immobilization of platelet antigen (MAIPA) methodologies in relation to DLX.

	DLX n (%)		
	Positive	Negative	Total
Pak-Lx			
Positive	28 (28)	14 (14)	42
Negative	11 (11)	47 (47)	58
Total	39	61	100
PIFT			
Positive	36 (36)	16 (16)	52
Negative	3 (3)	45 (45)	48
Total	39	61	100
MAIPA			
Positive	30 (30)	14 (14)	44
Negative	9 (9)	47 (47)	56
Total	39	61	100

### Analysis considering MAIPA as the reference for human platelet antigens antibodies

On comparing the Pak-Lx assay to MAIPA as the standard, an estimated sensitivity of 91 %, estimated specificity of 96 %, positive predictive and negative predictive values of 95 % and 93 %, respectively and an estimated concordance of 94 samples (94 %) showed agreement between the two techniques. In one case with IPR, the sensitivity of MAIPA failed to detect pan-reactivity for GPIa/IIa and in one case it failed to detect anti-5b. In another four cases, the Pak-Lx assay returned negative results, while the MAIPA showed positivity for the HLA.

On comparing PIFT to MAIPA as the standard, the estimated sensitivity was 95 %, estimated specificity was 82 %, positive predictive value was 81 %, negative predictive value was 96 %, and the estimated concordance was 88 %. In one case with IPR, the sensitivity of MAIPA failed to detect reactivity for GPIIb/IIIa, GPIa/IIa, GPIBIX or Beta2microglibulina, in one case, it failed to detect anti-5b and in three cases it failed to detect anti-HLA antibodies. In another two cases, the PIFT assay returned negative results, while the MAIPA showed positivity for HLA antibodies, one case of pan-reactivity.

The comparison between the Pak-Lx and PIFT methods regarding concordance with MAIPA resulted in a p-value of 0.114, indicating that there is no evidence of significant differences between the methods regarding their concordance with MAIPA. In ten cases of IPR, the sensitivity of the Pak-Lx assay failed to detect HLA antibodies. In another two cases, the PIFT assay returned positive results, while the Pak-Lx assay showed negativity (Table 3).

**Table 3:** Diagnostic measures calculated for platelet immunofluorescence test (PIFT) and Luminex platform-driven microsphere-based multiplex assays (Pak-Lx) in relation to monoclonal antibody-specific immobilization of platelet antigen (MAIPA).

Variable	Pak-Lx	PIFT
Prevalence (%)	44 (34–54)	44 (34–54)
Sensitivity (%)	91 (78–97)	95 (85–99)
Specificity (%)	96 (88–1.00)	82 (70–91)
Positive predictive value (%)	95 (84–99)	81 (67–90)
Negative predictive value (%)	93 (83–98)	96 (86–99)
Concordance rate (%) a	94 (87–98)	88 (80–94)

a The concordance between Pak-Lx versus PIFT: *p*-value = 0.114. The data is reported as the estimate (95 % confidence interval).

### Analysis considering DLX as the reference for human leukocyte antigens antibodies

Considering Pak-Lx compared to DLX as the standard, the estimated sensitivity was 72 %, estimated specificity was 77 %, positive predictive value was 67 %, negative predictive value was 81 %, and the estimated concordance was 75 %. On comparing PIFT to DLX as the standard, the estimated sensitivity was 92 %, estimated specificity was 74 %, positive predictive value was 69 %, negative predictive value was 94 %, and the estimated concordance was 81 %. In eleven cases of IPR, the sensitivity of the Pak-Lx assay failed to detect anti-HLA antibodies. In another fourteen cases, the DLX assay returned a positive result, while the Pak-Lx assay showed negativity.

The sensitivity and specificity of MAIPA compared to the DLX assay were 77 %, with a positive predictive value of 68 % and a negative predictive value of 84 %. The estimated concordance was 77 %. In nine cases of IPR, the sensitivity of the MAIPA assay failed to detect HLA antibodies. In another nine cases, the MAIPA assay returned a positive result, while the DLX assay showed negativity.

The comparison of the Pak-Lx assay, PIFT assay and MAIPA methods regarding concordance with the DLX assay resulted in p-values of 0.114 (Pak versus PIFT), 0.683 (Pak versus MAIPA), and 0.387 (PIFT versus MAIPA), indicating that there is no evidence of significant differences between the methods regarding their concordance with DLX (Table 4).

**Table 4:** Diagnostic measures calculated for platelet immunofluorescence test (PIFT) and monoclonal antibody-specific immobilization of platelet antigen (MAIPA) in relation to DLX.

Variable	Pak-Lx	PIFT	MAIPA
Prevalence (%)	39 (29–49)	39 (29–49)	39 (29–49)
Sensitivity (%)	72 (55–85)	92 (79–98)	77 (61–89)
Specificity (%)	77 (65–87)	74 (61–84)	77 (65–87)
Positive predictive value (%)	67 (50–80)	69 (55–81)	68 (52–81)
Negative predictive value (%)	81 (69–90)	94 (83–99)	84 (72–92)
Concordance rate (%) a	75 (65–83)	81 (72–88)	77 (68–85)

a The concordance between Pak-Lx versus PIFT: *p*-value = 0.114; Pak-Lx versus MAIPA: *p*-value = 0.683, and PIFT versus MAIPA: *p*-value = 0.387. Data reported as estimate (95 % confidence interval).

## Discussion

Three different methodologies were investigated in this study. Agreement was found for most cases between all the tests. The results obtained in the comparison between the MAIPA and Pak-Lx assays are consistent with the findings of Porcelijn et al. These authors showed that in 5 % of the samples tested, the Pak-Lx method detected the presence of antibodies not detected by MAIPA.^24^ Among the two samples showing positive results in the Pak-Lx assay, one had specificity for the anti-5b antibody and the other for an antibody targeting GPIa/IIa. Both displayed low average MFI values (1469 and 513), compared to optical densities of 0.121 and 0.033. In the detection of anti-HLA antibodies, four samples yielded negative results in the Pak-Lx assay compared to MAIPA. Our data differs from those obtained by Tao et al.^21^ who did not find significant differences between the Luminex assay and MAIPA. One of the reasons to explain this discrepancy in the current study is the low optical density presented by the samples (MAIPA between 0.309 and 0.506), and the antigenic composition of the HLA microspheres used did not include all possible reactive epitopes. Anti-HLA antibody samples were subsequently confirmed as positive by the DLX assay.

The comparison between the PIFT and MAIPA assays found five discrepant results. The antibodies detected reacted positively with low fluorescence signals, specifically anti-HLA (fluorescence between 6.9 and 20) and anti-5b (fluorescence of 14.3) in the PIFT assay. There were two negative results using PIFT that were positive using MAIPA; one case with anti-HLA antibodies and one pan reactivity case for GPIIb/IIIa. Comparing the PIFT and Pak-Lx assays, there were 12 discrepancies between the results, primarily due to differences in anti-HLA antibodies.

Differences in the detection of HLA antibodies were observed in some samples. However, as MAIPA is not intended for HLA antibody detection, the reactivity of the HLA bead in the PAK-Lx setting was compared to the reactivity of the corresponding bead in the DLX giving a concordance of 89 %. The most reliable technique for elucidating anti-HLA antibodies would be the analysis of single bead assays.

The PAK-Lx test offers significant advantages over MAIPA, with a shorter duration of approximately three hours eliminating the need for typed donor platelets or MoAb. Moreover, it only requires 10 μL of serum for antibody screening and identification. In contrast, MAIPA takes 6–8 h, demands skilled technicians, a typed donor platelet identification panel, glycoprotein-specific MoAbs, and larger quantities of patient serum.^24^ However, it is important to note that PAK-Lx currently lacks HPA-15 antibody identification beads, and confirming antibody specificity with the available beads may not always be feasible.

The adjusted ratio values in PAK-Lx and DLX, utilized by the software for positive or negative classification, act as indicators. Nevertheless, a meticulous examination of the results is imperative to identify weak antibodies. For instance, subtle antibody reactions that register as positive solely with homozygous beads, but not with heterozygous beads, might escape detection by the software and necessitate manual assessment of the signals.

The PIFT method, when employed to detect the presence of antiplatelet antibodies, proves to be a valuable tool in screening patients.^18^ Its speed, with a testing time of approximately two hours, and the requirement for a small amount of serum provide advantages compared to the MAIPA test.

We observed a greater discrepancy in the results of anti-HLA antibodies. This disparity can be attributed to the diversity of HLA antigens in the population, making it challenging to develop a panel or test capable of detecting all possible antibody specificities. This challenge is particularly evident in assays such as MAIPA and PIFT, which depend on known donor phenotypes. Individual specificity identification assays may be more suitable in overcoming this challenge. One way to mitigate this discrepancy would be to use the most common HLA antigens found in the population. This approach could help to reduce discrepancies observed between assays utilizing known donor antigens.

At present, platelet reference laboratories globally employ one or more methodologies to detect platelet antibodies.^16^ A prevalent approach involves the utilization of MAIPA combined with a modified enzyme-linked immunosorbent assay or commercial assays utilizing the Luminex platform. In the comparison of commercial assays with MAIPA, the Pak-Lx assay exhibited a false-negative rate of 6 % and a false-positive rate of 4.1 %.

In our panel of sera, the very rare specificities HPA-2a and GPIV were not included, and some other HPA specificities (HPA-1b, −2b, −3a, −5a) were present in a limited number of samples. Therefore, it will be necessary to conduct a more comprehensive evaluation of this assay, using a larger number of samples containing (rare) HPA specificities.

## Conclusions

The current study shows no significant differences in the concordance between the Pak-Lx and PIFT assays when compared to the MAIPA and DLX methods for detecting anti-HPA and anti-HLA antibodies, respectively. We conclude that both PAK-Lx and PIFT are sensitive and easily performed methods for screening platelet alloantibodies. However, in the case of weakly reactive antibodies or the detection of rare antibodies, confirmation of antibody specificity with MAIPA is recommended.

## Conflicts of interest

I declare that I am not subject to any kind of conflict of interest with participants or any other collaborator, directly or indirectly, for the development of the Research Project entitled ‘Platelet antibody detection assays: a single-laboratory comparison of MAIPA, PIFT, and microsphere-based multiplex assays Pak-Lx’ whose involved researchers are: Thiago Henrique Costa, Carolina Bonet Bub and José Mauro Kutner.

## Data Availability

Data underlying this study are available and can be utilized.
